# Mitigation of Fiber Print-Through During Replication of Carbon Fiber-Reinforced Polymer Mirrors

**DOI:** 10.3390/ma18030617

**Published:** 2025-01-29

**Authors:** Yuanpeng Fan, Lei Wei, Jian Pei, Lin Li

**Affiliations:** 1 Institute of Frontier and Interdisciplinary Science, Shandong University, Qingdao 266237, China; fanyuanpeng@mail.sdu.edu.cn (Y.F.); peijian980102@163.com (J.P.); 2 Space Optoelectronic Measurement and Perception Laboratory, Beijing Institute of Control Engineering, Beijing 100190, China

**Keywords:** carbon fiber-reinforced polymer (CFRP), mirror, fiber print-through (FPT), replication technique, roughness

## Abstract

Aiming at the phenomenon of fiber print-through (FPT) during the curing process of carbon fiber-reinforced polymer (CFRP) mirrors, this paper effectively mitigates FPT by improving the replication technique. This paper simulates and analyzes the causes of FPT generation and then improves the replication technique based on multiple explorations of the replication process. In the improved replication technique, a mold is designed to assist in the curing process of the mirrors and to further suppress the FPT on the surface by applying pressure during the curing process. In addition, the layup method, resin matrix, and curing equipment are also optimized and improved. The surface roughness of the CFRP mirrors before and after the process improvement is measured using an optical surface profilometer, and the roughness parameters such as the arithmetic average deviation, root mean square error, and maximum height are obtained. Measurement results show that the improved replication technique effectively mitigates FPT, the surface of the obtained CFRP mirror is smoother, and the application of pressure during curing can further reduce the roughness of the mirror surface.

## 1. Introduction

With the rapid development of space techniques and space optics, high-resolution remote sensing constellations have become a major development trend. The requirement of high resolution makes the aperture of space cameras larger and larger, which will inevitably increase the complexity of the camera structure and the launch cost. Most of the space cameras use reflective optical systems. Reducing the weight and design difficulty of the mirrors on the basis of guaranteeing the surface accuracy is an urgent problem to be solved at present.

Compared with traditional materials such as glass and metal, CFRP has advantages such as high specific stiffness, thermal stability, and strong designability [1,2]. CFRP is an ideal material to reduce costs. CFRP mirrors with high shape accuracy have become a hot research topic in the aerospace field. The replication technique is the most widely used method for preparing CFRP mirrors [3,4]. The replication technique for CFRP mirrors involves heating to fuse the resin matrix with the fibers; this process is called curing. During the curing process of mirror surface replication, FPT occurs on the mirror surface due to the fact that the contraction and expansion of the fibers and the resin matrix with temperature changes are not exactly consistent [5]. FPT can seriously affect the roughness of CFRP mirrors, and the mitigation of FPT through experimental or theoretical studies is an urgent problem in the research of CFRP mirrors.

Liu, Q.N. et al. [6] studied the influence of ply angle deviation on the surface accuracy of CFRP mirrors based on methods such as Monte Carlo and statistical analysis, and their research shows that the most effective way to improve surface accuracy is to reduce the standard deviation of the angle deviation. Arao, Y. et al. [7] also studied the influence of ply angle deviation on surface deformation through the method of stochastic analysis and found that the deformation was caused by the water absorption of the plies. The research of Cheng, L. et al. [8] indicates that ply angle deviation and ply thickness deviation can cause astigmatism and defocus in CFRP mirrors. Vincenti, A. et al. [9] conducted a theoretical study on the impact of ply angle errors on CFRP. Thompson, S.J. et al. [10] studied the combinations of plies with different angles and different numbers of layers and put forward some suggestions. Steeves, J. [11] discusses the effects of misalignments in ply orientation, uniform variations in ply thickness, and through-thickness thermal gradients on the shape of CFRP laminates. Núcleo Milenio de Formación Planetaria (NPF) in Chile has conducted a lot of work on the replication process of CFRP mirrors, and NPF points out that the FPT phenomenon can be suppressed by optimizing the layup method, coating, and adding additional layers of resin [12,13,14,15]. Studies of CFRP mirrors by the U.S. Air Force Research Laboratory and the University of New Mexico also indicate that adding an extra layer of resin to the surface and then polishing it can reduce surface roughness [16]. It can be seen from these studies that in addition to tight control of the thickness and the angle of the layup, coating and adding additional resin are currently effective ways to weaken the FPT phenomenon. However, the added resin layer can only be cured at room temperature and cannot be perfectly integrated with the high-temperature cured mirror surface, which will reduce the strength and thermal conductivity of the mirror. In addition, if the surface roughness after curing is poor, since the thickness of the coating is generally very thin, it will be difficult to obtain a smooth mirror surface even with coating.

This paper mitigates the FPT phenomenon through the improvement of the replication technique, which can reduce surface roughness while ensuring the mechanical and thermal performance of the mirror, which is more efficient than adding additional resin and coating. Firstly, this paper analyzes the causes of FPT generation. Then, this paper has made improvements to the replication technology in multiple aspects: (1) a mold was designed to assist in the curing and molding of the CFRP mirror; (2) through experiments, it was determined that applying a pressure of 0.2 MPa during the curing process can mitigate the FPT phenomenon; (3) through experimental comparison, an epoxy resin with a curing temperature of 130 °C was selected as the resin material; (4) through comparison, an autoclave was chosen as the equipment for curing and molding. Finally, this paper tests the roughness of the mirrors obtained before and after the improvement of the replication technique. The test results show that the improved replication technique can effectively mitigate FPT, and the application of pressure can make the mirror surface smoother.

## 2. Causes of FPT

FPT is an unavoidable phenomenon for CFRP mirrors during curing. During the curing of CFRP mirrors, due to the difference in the coefficients of thermal expansion (CTE) of the fibers and the resin, changes in temperature will lead to inconsistencies in the dimensions of their contraction and expansion, which will result in the geometric shape of the fiber bundles exposing the surface and causing a deterioration of the surface roughness, this phenomenon is referred to as FPT.

**Figure 1:**
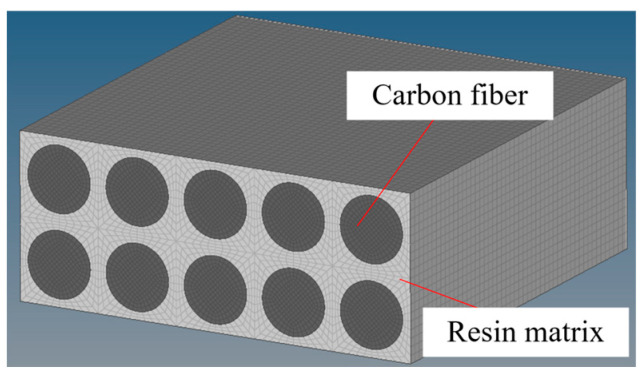
Finite element model for simulating the curing process.

**Figure 2:**
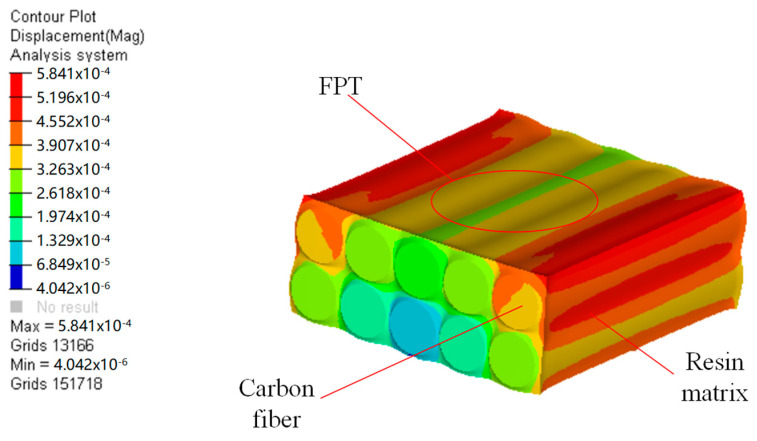
Deformation of CFRP after curing.

In order to understand FPT more intuitively and clearly, this paper simulates the curing process. Most of the resins used in CFRP are thermosetting resins, so the curing process can be simulated by reducing the temperature. Assuming that the curing temperature is 130 °C, the curing of CFRP can be simulated by using the process from 130 °C to 20 °C. The finite element simulation model is established as shown in Figure 1, and the parameters of the fibers and resin are shown in Table 1. The simulation results of the curing process are shown in Figure 2. From Figure 2, it can be seen that due to the inconsistency of the shape changes of the fibers and the resin, the shape of the fibers is revealed, and thus, the roughness will deteriorate.

## 3. Mandrel Manufacturing

The replication of CFRP mirrors first requires machining a mold of opposite curvature, which is generally referred to as a mandrel. The surface accuracy of the mandrel is generally required to be greater than the required surface accuracy of the CFRP mirror, so the mandrel usually uses optical materials.

The properties of commonly used optical materials are shown in Table 2. As can be seen from Table 2, the best specific stiffness is Be, followed by SiC and Zerodur. Be is not suitable because of its complicated processing and toxicity. SiC has an excellent specific stiffness, coefficient of thermal conductivity (CTC), and CTE, but the low yield of mirror blanks and the need for surface modification make it extremely expensive to machine, so it is also unsuitable as the material for making mandrels. Although Zerodur’s elastic modulus is slightly lower, the extremely low CTE ensures good performance during curing.

**Figure 3:**
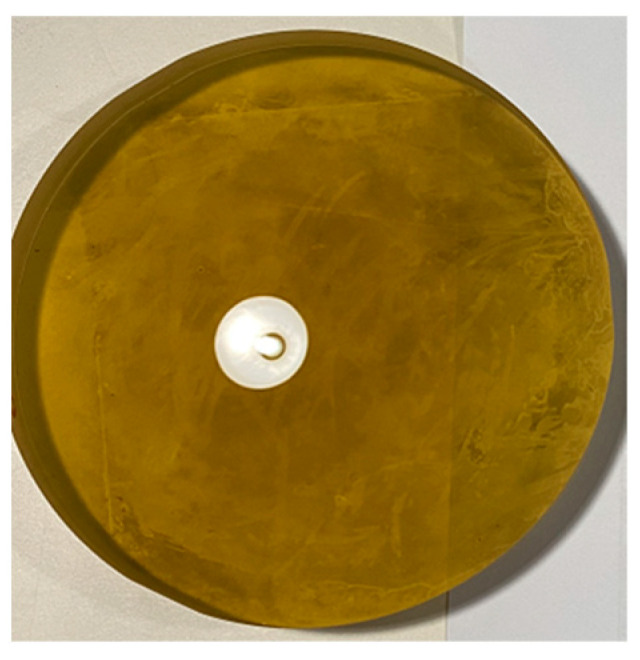
Mandrel manufactured by Zerodur.

The work of this paper is to research and improve the replication technique. The diameter of the mandrel is 130 mm, so using Zerodur as the material of the mandrel can meet the requirements. Zerodur is produced by Chengdu Guangming Optics Co., Ltd. Chengdu, China. The mandrel manufactured by Zerodur is shown in Figure 3; the surface of the mandrel is convex spherical, and the root mean square surface accuracy of this mandrel is λ/20 (λ = 632.8 nm).

It is important to note that the mandrel needs to be wiped in one direction using a dust free cloth moistened with alcohol to wipe the surface before each use, with the aim of cleaning it while avoiding scratching the surface. After cleaning, apply the release agent evenly on the surface of the mandrel to facilitate the separation of the mandrel and the sample after curing.

## 4. Improvements in Replication Technique

### 4.1. Process of Replicating Technique

**Figure 4:**
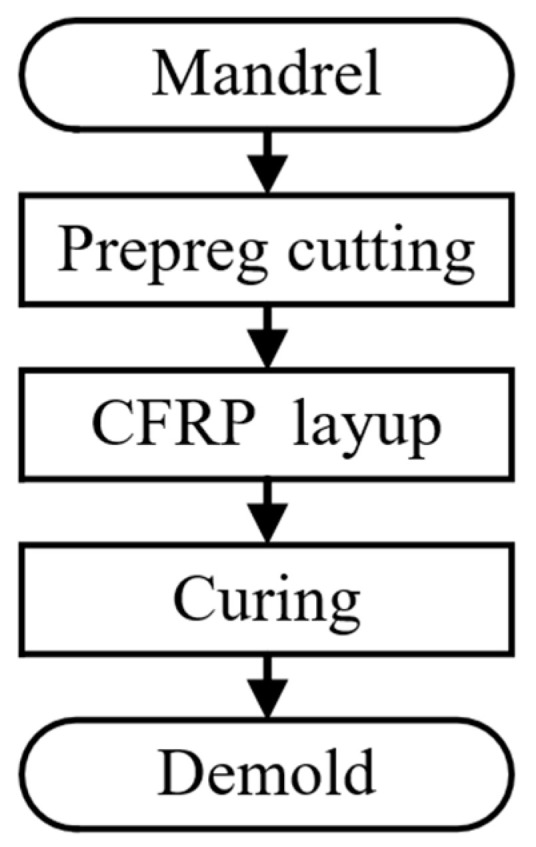
Basic process of replication technique.

The basic process of the CFRP mirror replication technique is shown in Figure 4. The first step in the replication technique is to manufacture a corresponding mandrel based on the desired CFRP mirrors. The second step is to cut the carbon fiber prepreg to the desired size. The third step is to lay the layers by hand. The fourth step is to put the sample into a vacuum bag and then put it in a heated container for curing. The fifth step is to separate the sample from the mandrel to obtain the CFRP mirror.

### 4.2. Replication of CFRP Mirrors Before Process Improvement

**Figure 5:**
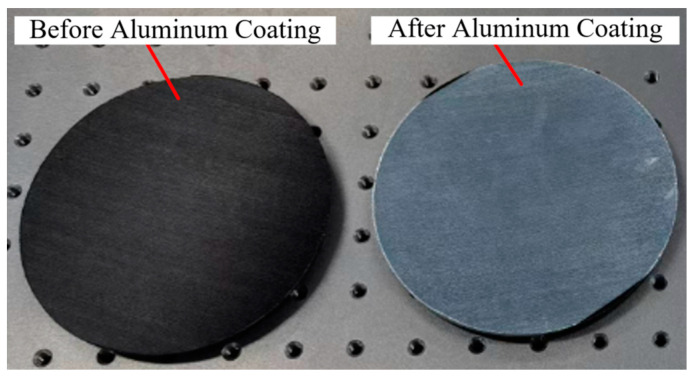
Samples obtained before replication technique improvement.

This study made some samples based on some existing research before improving the replication technique. The parameters of the replication process are shown in Table 3, and the obtained CFRP mirror and the CFRP mirror after aluminum coating are shown in Figure 5. The number “8” indicates that the ply sequence will be repeated 8 times in the entire layup structure. As can be seen from Figure 5, the surface of the sample has a very obvious FPT phenomenon, and the roughness is so poor that it cannot be used as a mirror. Even if the surface of the sample is coated with a layer of aluminum film, the roughness is still not greatly improved.

### 4.3. Improvement of Process Parameters

In order to mitigate the FPT, this paper improves the replication technology. The replication of CFRP mirrors is a complex process. During an attempt to increase the pressure, it was found that the surface of the sample became smoother. After 10 repeated trials, the conclusion was drawn that applying pressure during the curing process of CFRP mirrors can effectively mitigate FPT.

When manufacturing various CFRP products such as baffles, support trusses of space cameras, badminton racket frames, and fishing rod blanks, it was found that their product characteristics are quite sensitive to parameters such as the mold, curing pressure, layup method, resin matrix, and curing equipment. Therefore, this paper will improve these parameters, which will be introduced in detail below.

(1) Addition of auxiliary mold during curing

**Figure 6:**
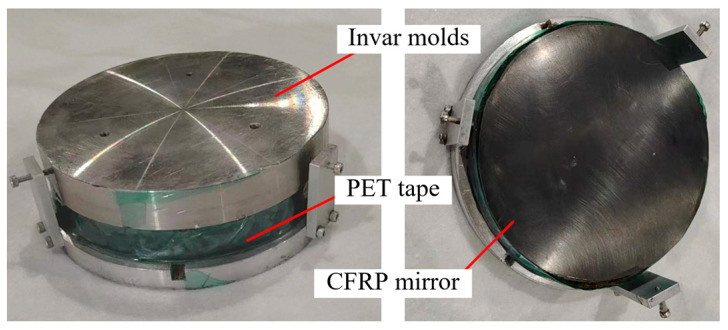
Auxiliary mold during curing.

The auxiliary mold can prevent the existence of gaps or lateral movement between the sample and the mandrel during curing. At the same time, the auxiliary mold can also make the pressure applied to the sample more uniform during the curing and molding process. There are mainly three principles for the design of the auxiliary mold: First, the curvature of the mold surface should be the same as that of the mirror. Second, the material of the mold should have a CTE similar to that of Zerodur. Third, the diameter of the mold should be larger than that of the sample. The auxiliary mold designed according to these three principles in this paper is shown in Figure 6. The mold is mainly composed of an upper cover, a chassis, and a tooling. The material of the mold is Invar steel, which has a CTE similar to that of Zerodur. After adding the mold, the overall envelope size is φ150 mm × 60 mm.

In addition, before placing the mold containing the CFRP mirror to be cured into a vacuum bag, it needs to be sealed with high-temperature-resistant polyethylene terephthalate (PET) tape, which can effectively prevent the resin from flowing out during the curing process.

(2) Application of pressure during curing

Taking advantage of the fluidity of the resin matrix at high temperatures, pressure is applied to make the sample and the mandrel surface fit more closely during curing, thereby obtaining a low-roughness CFRP mirror. Pressure during curing needs to go through the process of increasing, holding, and decreasing pressure. If the pressure is too small, the weakening of the FPT will not be obvious, and if the pressure is too high, the mandrel will break.

**Figure 7:**
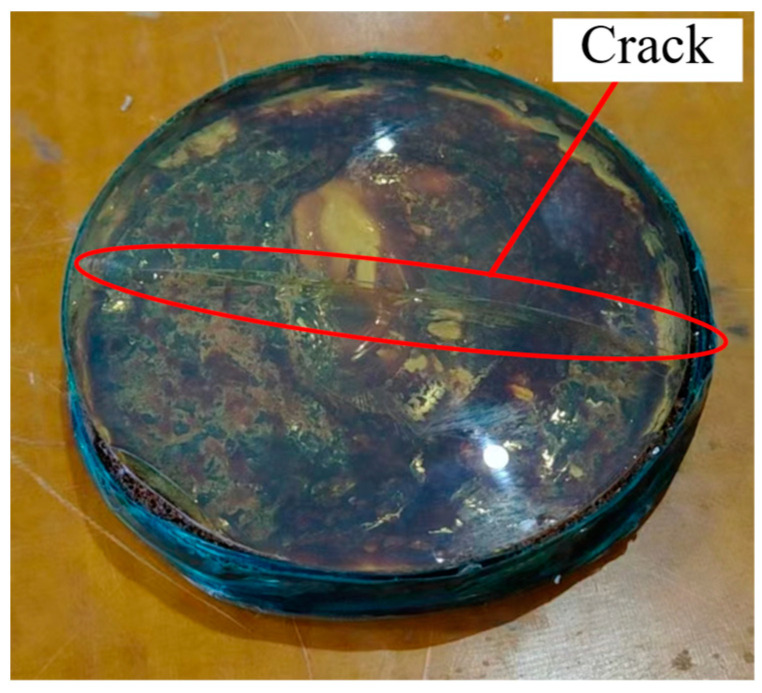
Excessive pressure causes a crack in the mandrel.

**Figure 8:**
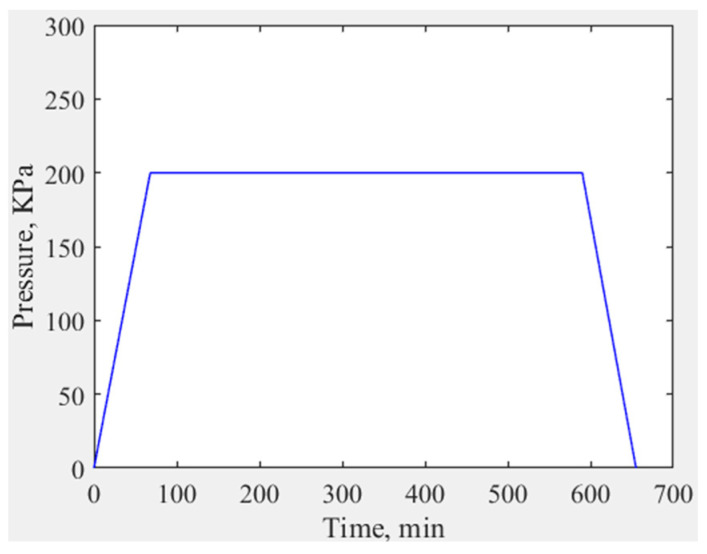
Pressure control curve during curing.

The autoclave is connected to a vacuum pump. After placing the CFRP mirror sample, the air is evacuated to a specified value, and then inert gas is introduced into the autoclave to increase the pressure. As shown in Figure 7, in a previous experiment, when the pressure exceeded 0.2 MPa, a crack appeared in the middle of the mandrel used. This indicates that for this mandrel, 0.2 MPa is the upper limit of the applied pressure. Therefore, this paper will discuss the samples obtained under peak pressures of 0 and 0.2 MPa. The pressure control curve is shown in Figure 8. In the first hour, the pressure inside the autoclave will rise to 0.2 MPa. Then, after maintaining this pressure for 9 h, a one-hour pressure-reducing process will commence.

(3) Improvement of the layup method

Different angles and thicknesses of layups are paired with each other to enable different mechanical and thermal properties. In order to obtain a quasi-isotropic CFRP mirror, the layup method is usually used 45° apart to achieve uniform distribution of mirror stress, and the symmetrical layup method is used to make the performance of the CFRP mirror more stable. NPF’s research points out that when the same layers are stacked together, the fibers can be aligned more efficiently, thus mitigating the FPT to some extent [10]. However, experience shows that stacking too many of the same layups can affect the strength of the surface.

This paper refers to the layup method used by NPF but reduces the number of identical layups at the mirror. The layup angle is [90°4/0°2/45°/−45°/−45°/−45°/−45°]s, the letter “s” represents a symmetric layup, where the laminates on both sides are completely symmetric with respect to the middle. The thickness of each layer is 0.1 mm by hand layup, and the error of layup angle is ±0.5°.

(4) Improvement of resin matrix

Fiber strands are obtained after high-temperature oxidation exceeding 2000 °C and have excellent high-temperature resistance, so the fibers will not be greatly deformed during curing. The curing temperature of CFRP is generally in the range of 80 °C to 200 °C, with different curing temperatures for different types of resin matrix. In general, resins that require high-temperature curing are more fluid than those that require low-temperature curing.

In order to prevent defects such as porosity, and to obtain CFRP mirrors with low roughness, the resin is required to have good flowability. However, the curing temperature cannot be too high. Because the higher the temperature, the more the resin will shrink during curing, and the roughness of the CFRP mirror surface will increase.

**Figure 9:**
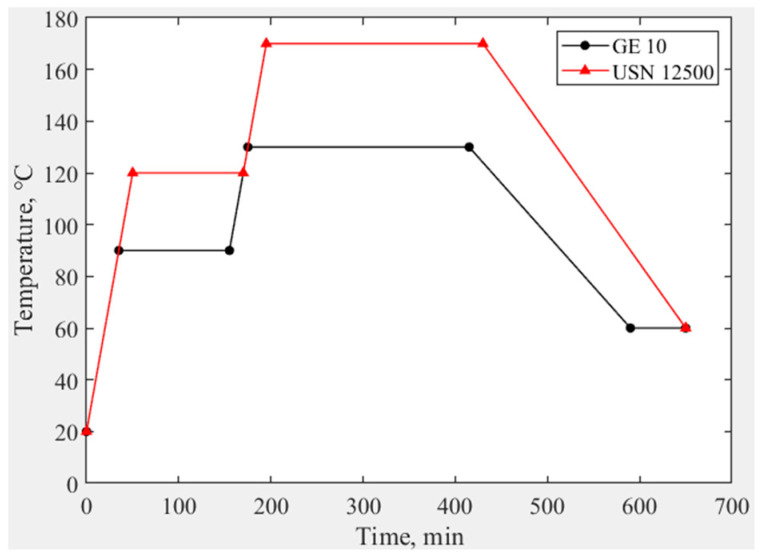
Temperature control curve of resin during curing.

**Figure 10:**
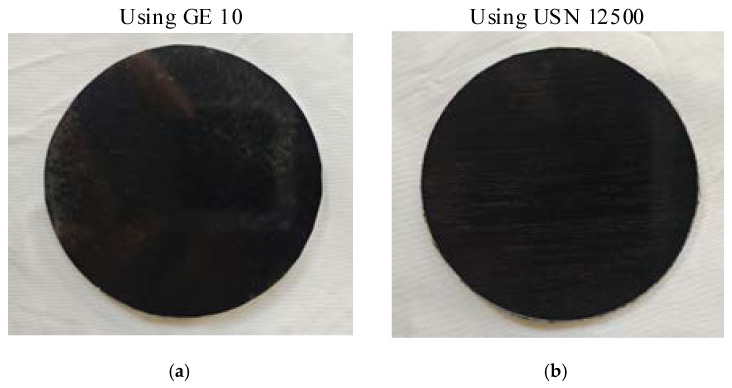
CFRP mirrors with different resin matrices. (**a**) Using GE 10. (**b**) Using USN 12500.

This paper compares the replication effects of epoxy resin (brand: GE 10) with a curing temperature of 130 °C and epoxy resin (brand: USN 12500) with a curing temperature of 170 °C, which are commonly used in the process. All process parameters are the same except for the type of resin and the required curing temperature. The need for temperature during curing goes through the process of warming, holding, and cooling; the temperature control curve is shown in Figure 9, and the samples obtained are shown in Figure 10. As can be seen from Figure 9, within the first three hours, the temperature inside the autoclave will increase in two stages. Then, after maintaining the maximum temperature for four hours, a three-hour cooling process will begin. As can be seen from Figure 10, there is a significant difference in the surface roughness of the two samples. The surface roughness of the sample using USN 12500 is worse after curing. Therefore, this paper will use GE 10 to replicate CFRP mirrors. The two resin matrixes are produced by Harbin FRP Institute, and the institute is located in Harbin, China.

(5) Selection of curing equipment

Temperature and pressure need to be controlled during the curing process, and there are two types of equipment available: an autoclave and a pressure oven. The pressure oven has a lower cost, but the autoclave offers better control over temperature and pressure. Moreover, the autoclave has a relatively large internal space, ensuring that curing and molding can take place under uniform conditions at every position, making it more suitable for mass-producing products. In order to obtain CFRP mirrors with low roughness after curing, this paper will choose to use an autoclave as the curing equipment.

**Figure 11:**
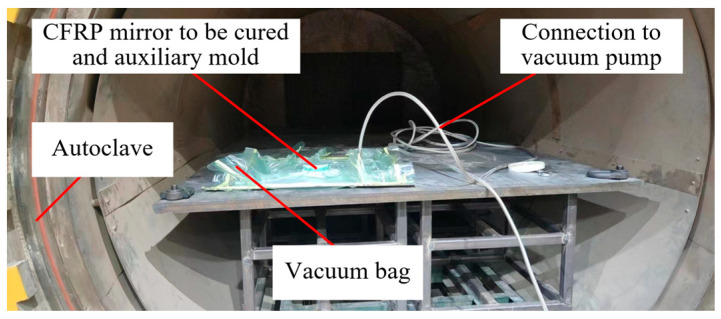
Autoclave used in curing process.

As shown in Figure 11, the auxiliary mold containing the sample to be cured needs to be placed into a vacuum bag, and then a vacuum pump is used to evacuate the vacuum bag. This removes air from the sample and prevents air pockets in the cured sample. Finally, the autoclave is closed to heat up and pressurize.

In summary, the parameters of the improved replication process are shown in Table 4.

In summary, this paper has completed the improvement of the replication process. In this paper, an auxiliary mold was designed for the curing and molding process of the CFRP mirror. This mold can prevent the movement of the sample and make the applied pressure more uniform. Through experiments, it was determined that the upper limit of the pressure applied to the sample during the curing process is 0.2 MPa. Therefore, two groups of experiments, one without pressure and the other with a pressure of 0.2 MPa, were carried out in this paper. By means of experimental comparison, it was determined that an epoxy resin with a curing temperature of 130 °C is more suitable for the replication process of the CFRP mirror. The autoclave, which has higher precision in controlling temperature and pressure, is selected as the equipment for curing and molding.

## 5. Measurement Results

**Figure 12:**
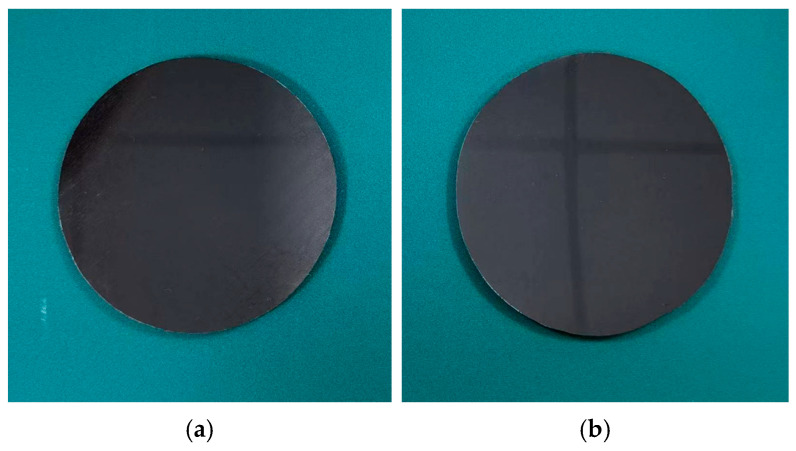
Replicated CFRP mirrors. (**a**) No curing pressure is applied. (**b**) Applying 0.2 MPa curing pressure.

The improved process is used to obtain mirror samples with no pressure and 0.2 MPa pressure applied during curing, as shown in Figure 12. After visual inspection, the surface smoothness of the sample obtained after the improved process is significantly better than that of the sample obtained before the improved process in Figure 5, and the reflection effect of the sample obtained by applying pressure is better than that without applying pressure.

Whether the mitigation of FPT is effective can be evaluated by roughness. A low surface roughness value indicates that the degree of undulation and unevenness on the CFRP mirror is small, meaning a high level of surface smoothness, and thus, FPT is effectively alleviated.

**Figure 13:**
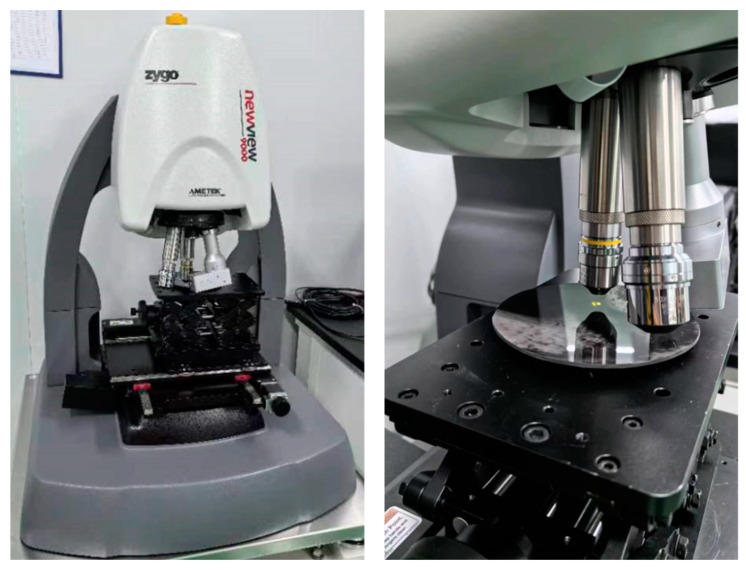
Roughness detection using optical surface profilometer.

**Figure 14:**
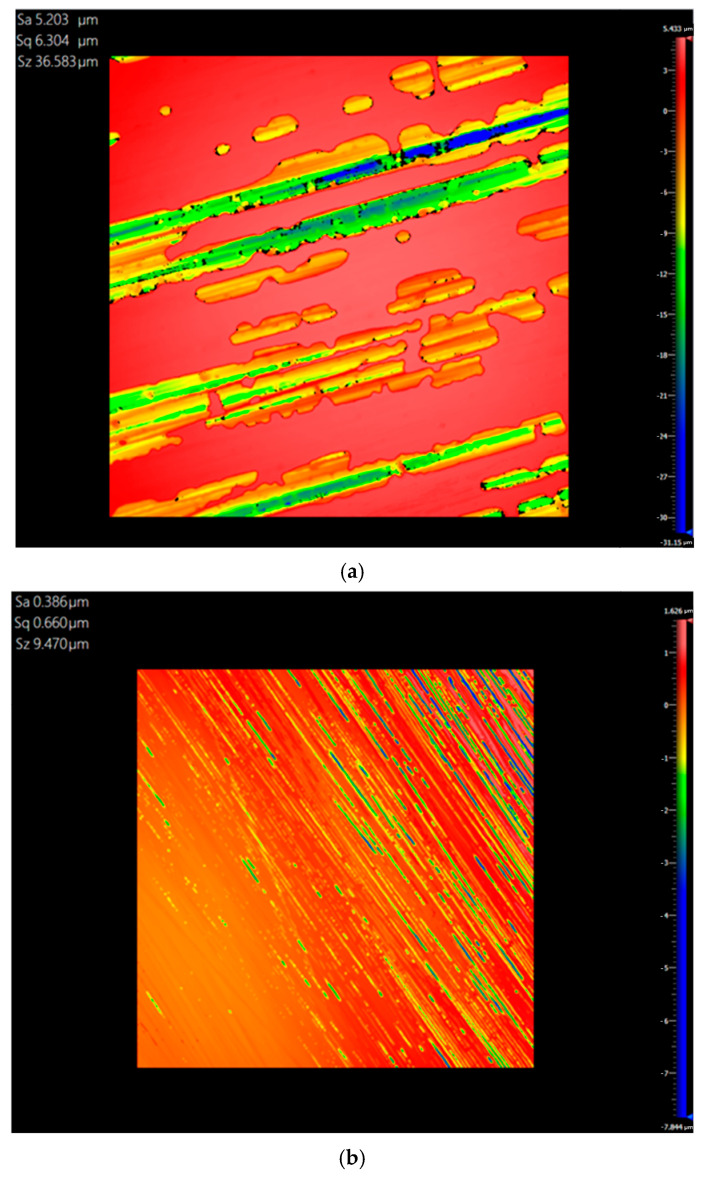

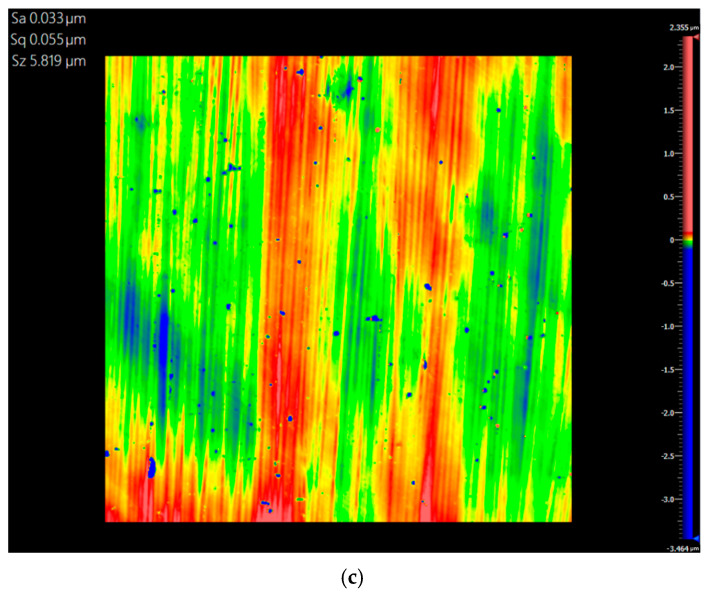
Deformation contours of surface roughness. (**a**) Sample result of the sample obtained before process improvement. (**b**) Sample result of the sample obtained without curing pressure after process improvement. (**c**) Sample result obtained by applying 0.2 MPa curing pressure after process improvement.

As shown in Figure 13, this paper uses the optical surface profilometer ZYGO NewView 9000 to detect the surface roughness of CFRP mirrors. ZYGO NewView 9000 is manufactured by ZYGO Corporation. The company is headquartered in Connecticut, USA. We measured the surface roughness of three samples obtained before and after the improvement of the replication technique. The sizes of the detection areas are almost the same. The measurement results are shown in Figure 14 and Table 4.

Arithmetic mean deviation is the arithmetic mean of the absolute values of the vertical coordinates within the sampling area and is expressed as Sa in the measurement results. The root mean square error is the root mean square value of the vertical coordinate over the sampling area and is expressed as Sq in the measurement results. The maximum height is the height of the sum of the maximum peak height and the minimum valley depth in the sampling area and is expressed as Sz in the measurement results.

As can be seen from the measurement results in Table 5, the surface roughness of the CFRP mirror obtained by the improved process is significantly lower than that of the samples before the process improvement. The surface roughness of the CFRP mirror obtained under pressure during the curing process is significantly lower than that of the samples without pressure applied. Therefore, both the improvement of the process and the application of pressure can mitigate the impact on FPT.

## 6. Conclusions

This paper investigates how FPT during curing can be mitigated by improvements in the replication technique for CFRP mirrors. Firstly, this paper analyzes the causes of FPT and introduces the production of the mandrel necessary for the replication technique. Then, this paper improves the replication technique in terms of the design of auxiliary mold, the application of pressure during curing, the change in the layup method, the selection of the resin matrix, and the use of curing equipment. Finally, an optical surface profilometer is used to detect the roughness parameters such as the arithmetic mean deviation, root mean square error, and maximum height of the samples before and after the process improvement. The measurement results show that the surface of the CFRP mirror obtained by the improved replication technique is smoother, and the application of pressure during curing makes the roughness lower, and FPT is effectively mitigated. How to optimize the auxiliary mold so that the mandrel can withstand greater pressure and explore the optimal value of pressure is our next work plan. This study hopes to promote or serve as a reference for further research on CFRP mirrors.

The prospects of CFRP mirror replication technology are extremely broad, and the future research directions are clear and crucial. At the material level, continuous improvement of new carbon fiber and resin systems is needed to optimize the performance of CFRP. In terms of the process, there is an urgent need to solve problems such as improving surface shape accuracy, shortening the replication cycle, and conquering the replication of complex shapes. The application potential of CFRP mirrors is enormous. In the aerospace field, they can be used in space telescopes and satellite remote sensing. In astronomical observations, they can serve large ground-based and radio telescopes. Fields such as laser communication, medical imaging, and high-end optical instruments will also benefit from the development of CFRP mirror replication technology and achieve leapfrog development.

## Figures and Tables

**Table 1:** Simulation parameters for the curing process.

Parameters	Elastic Modulus (MPa)	Poisson’s Ratio	Density (g/cm^3^)	CTE
Fiber	330,000	0.27	1.8	−8.3 × 10^−7^
Resin	2000	0.33	1.6	2.8 × 10^−5^

**Table 2:** Properties of commonly used optical materials.

Parameters	Be	Al	SiC	Fused Silica	Zerodur
Density (g/cm^3^)	1.85	2.7	3.05	2.2	2.5
Elastic Modulus (GPa)	280	69	400	67	92
CTC (W/(m K))	157	220	185	1.38	1.2
CTE (10–6/K)	11.4	23.9	2.5	0.55	0.027

**Table 3:** Parameters of the replication process of CFRP mirrors.

Replication Process	Parameters
Layup method	[0°/45°/90°/−45°]_8_
Curing pressure	0
Resin	Epoxy (brand: USN 12500)
Curing temperature	170 °C
Curing equipment	Oven

**Table 4:** Parameters of the improved replication process.

Replication Process	Parameters
Layup method	[90°4/0°2/45°/−45°/45°/−45°]s
Curing pressure	0.0 and 0.2 MPa
Resin	Epoxy (brand: GE 10)
Curing temperature	130 °C
Curing equipment	Autoclave

**Table 5:** Measurement results of surface roughness.

Surface Roughness	Arithmetic Average Deviation (μm)	Root Mean Square Error (μm)	Maximum Height (μm)
Sample before process improvement	5.203	6.304	36.583
Sample without pressure after process improvement	0.386	0.660	9.470
Sample with 0.2 MPa pressure after process improvement	0.033	0.055	5.819

Note: Roughness error of about 0.005 μm.

## Data Availability

The raw/processed data required to reproduce these findings cannot be shared at this time as the data also form part of an ongoing study.
